# Iodine Content of Wild and Farmed Seafood and Its Estimated Contribution to UK Dietary Iodine Intake

**DOI:** 10.3390/nu14010195

**Published:** 2021-12-31

**Authors:** Matthew Sprague, Tsz Chong Chau, David I. Givens

**Affiliations:** 1 Institute of Aquaculture, Faculty of Natural Sciences, University of Stirling, Stirling FK9 4LA, UK; 2 Institute for Food Research and Nutrition, University of Reading, Agriculture Building, P.O. Box 2376, Earley Gate, Reading RG6 6AR, UK; tcchau603@gmail.com (T.C.C.); d.i.givens@reading.ac.uk (D.I.G.)

**Keywords:** iodine, seafood consumption, wild fish, aquaculture, public health

## Abstract

Iodine is an important nutrient for human health and development, with seafood widely acknowledged as a rich source. Demand from the increasing global population has resulted in the availability of a wider range of wild and farmed seafood. Increased aquaculture production, however, has resulted in changes to feed ingredients that affect the nutritional quality of the final product. The present study assessed the iodine contents of wild and farmed seafood available to UK consumers and evaluated its contribution to current dietary iodine intake. Ninety-five seafood types, encompassing marine and freshwater fish and shellfish, of wild and farmed origins, were purchased from UK retailers and analysed. Iodine contents ranged from 427.4 ± 316.1 to 3.0 ± 1.6 µg·100 g^−1^ flesh wet weight (mean ± SD) in haddock (*Melanogrammus aeglefinus*) and common carp (*Cyprinus carpio*), respectively, being in the order shellfish > marine fish > freshwater fish, with crustaceans, whitefish (Gadiformes) and bivalves contributing the greatest levels. Overall, wild fish tended to exhibit higher iodine concentrations than farmed fish, with the exception of non-fed aquaculture species (bivalves). However, no significant differences were observed between wild and farmed Atlantic salmon (*Salmo salar*), rainbow trout (*Oncorhynchus mykiss*), and turbot (*Psetta maxima*). In contrast, farmed European seabass (*Dicentrarchus labrax*) and seabream (*Sparus aurata*) presented lower, and Atlantic halibut (*Hippoglossus hippoglossus*) higher, iodine levels than their wild counterparts, most likely due to the type and inclusion level of feed ingredients used. By following UK dietary guidelines for fish consumption, a portion of the highest oily (Atlantic mackerel, *Scomber scombrus*) and lean (haddock) fish species would provide two-thirds of the weekly recommended iodine intake (980 µg). In contrast, actual iodine intake from seafood consumption is estimated at only 9.4–18.0% of the UK reference nutrient intake (140 µg·day^−1^) across different age groups and genders, with females obtaining less than their male equivalents.

## 1. Introduction

Iodine is an essential trace element with an important role in the regulation of vertebrate development and metabolism, being necessary for the biosynthesis of the thyroid hormones thyroxine and tri-iodothyronine [1,2,3]. Deficiencies in iodine intake can result in a variety of known human health disorders that affect the thyroid (goitre), as well as cognitive development and function that can affect an individual at any stage during life from foetus to adulthood [1,4,5]. Consequently, health organisations worldwide have set reference nutrient intake (RNI) levels to ensure that populations receive an adequate intake to prevent public health problems. In the UK, the current RNI for adults is 140 µg·day^−1^ [6], slightly lower than the 150 µg·day^−1^ RNI for adults and ≥200 µg·day^−1^ RNI for pregnant and lactating women advocated by the World Health Organisation (WHO) and other health authorities [7,8,9,10]. These allowances are mostly expected to be satisfied through dietary consumption. Nevertheless, in the UK, as well as Western Europe in general, there is some concern that iodine intake has become mild to moderately inadequate, especially among more vulnerable groups such as young children and women of reproductive age, despite improvements in nutrition [4,5,11,12,13,14].

Milk and dairy products tend to be the main contributing sources to dietary iodine intake in most Western countries including in the UK [15,16,17,18,19], although the iodine contents of these foods are largely dependent upon the drinking water and fortification of animal feed among other factors [20,21,22,23]. In contrast, iodine (as iodide) in aquatic environments is naturally incorporated into organisms, especially marine, at high concentrations [24,25]. Seafood, therefore, is commonly regarded as being the richest source of iodine in the human diet in addition to supplying other essential nutrients beneficial to human health and development. Indeed, many health advisory bodies and government agencies advise consuming at least two portions of fish per week as part of a healthy-balanced diet [26,27,28,29]. Thus, routine seafood consumption has the potential to facilitate populations achieving a sufficient iodine intake. 

In recent years, the volume of seafood produced through farming (aquaculture) has grown considerably such that it now supplies more for the human table market than that provided by wild capture fisheries [30]. This growth has been driven largely by the continual increase in the global population, resulting in a rise in demand for both high-value farmed species such as Atlantic salmon (*Salmo salar*), European seabass (*Dicentrarchus labrax*) and marine shrimp alongside traditionally farmed species such as carps and tilapias [31]. However, one of the major challenges associated with the success of aquaculture has been the sourcing of suitable feed ingredients to supply the rapidly growing industry. This has resulted in changes to feed formulations, especially for marine carnivorous species, shifting from the traditional inclusion of the finite and limited marine ingredients, fish meal and fish oil, to alternatives such as terrestrial plant-based raw materials that are generally considered more sustainable [32]. Subsequently, a reduction in the levels of some beneficial nutrients have been observed in both feeds and flesh [33,34,35], leading to the question of whether farmed fish can supply sufficient levels of essential nutrients to the human consumer without revision to current guidelines for fish consumption [36].

Although the iodine contents of seafood have been studied (e.g., [15,37,38,39,40,41,42]), they are generally limited to only a few species most commonly consumed. Food composition tables such as *McCance and Widdowson’s The Composition of Foods* provide important nutrient information in assessing the health and nutritional status of populations [43], although they must be maintained and updated in order for the data to remain relevant [44]. The UK’s dataset on popular fish and fish products was last updated in 2013 [45]. In the intervening period the variety of fish available to the consumer has increased and the nutrient compositions of farmed species have changed. Therefore, the present study sought to analyse and compare the iodine contents of 95 seafood products, consisting of marine and freshwater fish and shellfish (crustaceans, bivalves and cephalopods) of both wild and farmed origins, available to UK consumers and apply the data to estimate current iodine intake from seafood consumption.

## 2. Materials and Methods

### 2.1. Sample Collection and Preparation

A total of 95 different seafood samples comprising fresh and/or frozen fish and shellfish (crustaceans and molluscs) of wild and/or farmed origin were purchased from a variety of UK retailers (supermarkets, fishmongers, online retailers) between January 2016 and December 2019 (See Table 1 for details). Samples of the same species (minimum of 3) were obtained at different times as well as from different retailers, where available, to minimise the risk of sampling from the same individual fish or catch/harvest. On arrival to the laboratory, samples were thawed, where required, and skinned and boned or shelled, where necessary, leaving the main edible flesh which was subsequently homogenised to a smooth pâté using a blender mixer (Blixer^®^ V.V., Robot-Coupe, Vincennes, France). All samples were raw unless otherwise stated. Large whole fish, or cuts thereof, were generally determined on an individual basis, whereas smaller-sized fish such as European anchovies (*Engraulis encrasicolus*) and sprats (*Sprattus sprattus*) and shellfish purchased at the same time were analysed on a pooled basis. A sub-sample of the homogenate (~5–10 g) was weighed out and oven-dried at 110 °C for 20 h [46], with the remaining sample stored at −20 °C for further study. The dried sample was reweighed and ground to a fine powder with the moisture content noted in order to express results on a wet weight (ww) basis. Dried samples were stored in darkness under vacuum in a desiccator until shipped to the University of Reading for iodine analysis. Sample identities (i.e., species, wild/farmed location, etc.) were based on the product/label information available at the time of purchase. Ethical approval for this study was granted by the Animal Welfare and Ethical Review Body (AWERB) atodine in food- and dietary supplemen the University of Stirling (AWERB/167/208/New Non ASPA).

### 2.2. Iodine Analysis

Total iodine contents were determined using inductively-coupled plasma mass spectrometry (ICP-MS). Samples (100–500 mg) were analysed following an alkaline extraction using 2.74 M tetramethyl ammonium hydroxide (TMAH; Acros Organics, NJ, USA) essentially according to the method of Fecher et al. [49]. Briefly, samples plus 5 mL of ultra-pure water plus 1 mL TMAH were vortex mixed to ensure homogeneity and placed in an oven at 90 °C ± 5 °C for 3 h. After cooling, dilution with ultra-pure water and centrifugation (5000× *g*, 12 min) the supernatant was filtered through a 0.45 μm membrane filter diameter ~33 mm (Fisher, Pittsburgh, PA, USA) and analysed by ICP-MS (iCAP Q, Thermo Scientific Inc., Waltham, MA, USA). All apparatus used was washed with 6 M nitric acid and then soaked for at least 24 h in ultra-pure water.

Analyses were performed in triplicate with the coefficient of variation amongst triplicate results typically being less than 10 percent with higher values leading to repeat analyses. A certified reference material (Fish muscle ERM-BB422; Institute for Reference Materials and Measurements (IRMM), Geel, Belgium) was included with each sample batch to assess the integrity of the sample analysis procedure. Instrument calibration standards (0–100 µL^−1^) were prepared from dilution of a stock solution of potassium iodide with TMAH to give a concentration equivalent to the samples. The limit of quantification (LOQ) was 0.04 mg·kg^−1^ dry weight. A reference calibration mix (DG-IC Calibration Mix multi-ion reference solution, BO3, Primag; Crawford Scientific, Lanarkshire, UK) was additionally used. The iodine concentrations were expressed on wet basis (i.e., as consumed). 

### 2.3. Seafood Contribution to Recommended Intake Levels

The consumption of a 140 g seafood serving, based on UK guidance [28], was used to calculate its contribution to a recommended weekly iodine intake of 980 µg was calculated according to the 140 µg·day^−1^ reference nutrient intake (RNI) for UK adults [6]. Estimation of the current UK iodine intake from seafood consumption was determined using the iodine results from the present study combined with available data on seafood consumption calculated according to age groups (1–5, 6–10, 11–18 and 19–64 years) from disaggregated seafood data from years 1 to 8 combined of the UK’s National Diet and Nutrition Survey (NDNS) [50]. Seafood species were matched accordingly to species consumption data, with processed products (e.g., fish fingers) paired using data from identified species. However, where no relevant fit could be made (i.e., fish pie with no species stated), appropriate data from *McCance and Widdowson’s The Composition of Foods* integrated dataset were applied [43].

### 2.4. Statistical Analysis

Statistical analyses were used to compare mean iodine concentrations between wild fish and their respective farmed counterparts. Data were analysed using Minitab^®^ v18.1 statistical software package (Minitab Inc., State College, PA, USA). Data were assessed for normality with the Kolmogorov–Smirnov test and for homogeneity of variances by Bartlett’s test together with the examination of residual plots and, where necessary, transformed by arcsine or natural logarithm. Data were compared by one-way analysis of variance (ANOVA) with multiple comparisons made using Tukey’s post hoc test. A significance of *p* < 0.05 was applied to all statistical tests performed.

## 3. Results and Discussion

### 3.1. Iodine Content of Seafood

**Figure 1:**
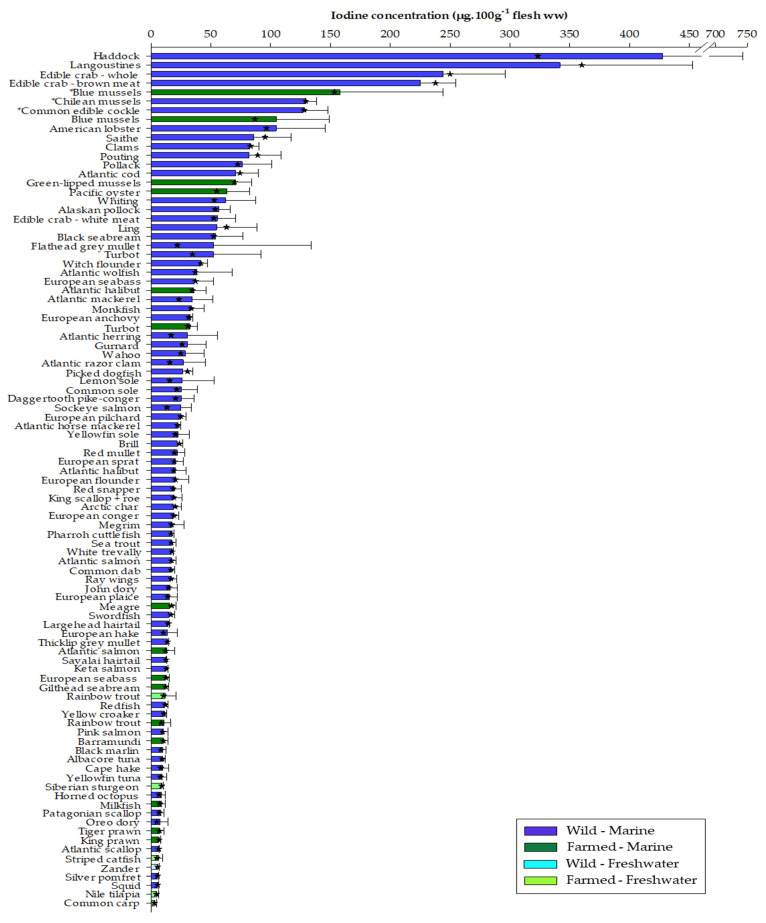
Iodine concentrations (µg·100 g^−1^ edible flesh, ww, mean ± SD) of 95 seafood samples of wild and or farmed marine or freshwater origin analysed in the current study. Samples ranked in descending order. 🟊 indicates median iodine value. All samples were analysed raw unless denoted by *. Refer to Table 1 for further sample information.

The iodine contents of the 95 seafood samples analysed in the present study are presented in descending order in Figure 1. Iodine concentrations (mean ± SD) ranged from 2.97 ± 1.58 µg·100 g^−1^ flesh ww in common carp (*Cyprinus carpio*) to 427.4 ± 316.1 µg·100 g^−1^ flesh ww in Atlantic haddock (*Melanogrammus aeglefinus*). Overall, the mean and median iodine values of the samples studied were comparatively uniform with each other, generally indicating that the variation in iodine levels can be considered as low (see Table S1 for values). However, large variations in iodine contents are known to occur between individuals from the same species [1,38,40,51,52] and the present study is no exception. In particular, haddock (427.4 and 323.3 µg·100 g^−1^ flesh ww, mean and median, respectively), flathead grey mullet (*Mugil cephalus*, 52.2 and 21.9 µg·100 g^−1^), wild turbot (*Psetta maxima*, 52.0 and 34.6 µg·100 g^−1^), Atlantic mackerel (*Scomber scombrus*, 34.4 and 23.3 µg·100 g^−1^), Atlantic herring (*Clupea harengus*, 30.4 and 16.8 µg·100 g^−1^), Atlantic razor clam (*Ensis ensis*, 26.9 and 15.7 µg·100 g^−1^), lemon sole (*Microstomus kitt*, 26.1 and 15.5 µg·100 g^−1^), and sockeye salmon (*Oncorhynchus nerka*, 24.9 and 13.3 µg·100 g^−1^) all exhibited sizeable differences between their respective mean and median values. There are several factors that may account for differences between individuals of the same species. Location (fishing ground) may be influential in accounting for any intra-species variability as seawater iodine concentrations, present as either iodate (IO_3_^−^) and iodide (I^−^) or minor amounts of dissolved organic iodine, are reported to vary both with depth and geographical location [24,25]. Thus, identifying fishing grounds allows for direct comparisons between data to be made while minimising potential misinterpretation. For example, Nerhus et al. [40] found that both Atlantic cod (*Gadus morhua*) and pollack (*Pollachius pollachius*) from the North Sea contained lower iodine levels than those fished within the Norwegian and Barents seas and/or Norwegian fjords. Based on the current findings, the 76.1 ± 24.7 µg·100 g^−1^ measured in pollack, all labelled as being caught in the North Sea (FAO Fishing area 27, subarea IV), is within range, albeit slightly lower, than the mean value reported for other North Sea pollack (210 µg·100 g^−1^) [40]. However, if the current value is compared to the substantially higher mean value of 790 µg·100 g^−1^ reported for pollack from all North East Atlantic fishing areas (FAO27) [40], then this difference could potentially be falsely interpreted as being attributed to an error such as during sample analysis. Cod from the present study, on the other hand, were labelled as being caught in either the Barents, Norwegian and North Seas as well as Icelandic grounds (FAO27 subareas I, II, IV and V, respectively), although the iodine level (70.7 ± 19.0 µg·100 g^−1^) more closely resembled the value observed by Nerhus et al. [40] for North Sea cod (96 µg·100 g^−1^) than either the Barents or Norwegian seas (400 and 250 µg·100 g^−1^, respectively) or from the mean value of all three fishing grounds (190 µg·100 g^−1^). Such variation in the iodine contents from the same species between locations may also be influenced by the available food supply [51]. The nutritional and physiological status of an individual fish is predominantly influenced by season, which affects both food availability and reproductive status [53]. Delgado et al. [38] found that mackerel and sardines (*Sardina pilchardus*) purchased from Portuguese markets tended to exhibit higher iodine contents in summer/autumn months compared to winter/spring. Similarly, Nerhus et al. [40] observed higher iodine concentrations in haddock sampled during the latter part of the year, which they attributed to a repletion of elements following the April-May spawning period. Samples from the present study were purchased at different times of the year where possible, although some species only appear on the market based on their seasonal availability. Moreover, wild species that are found typically on UK shelves all-year round, such as cod, mackerel, herring and all Pacific salmon species, will have almost certainly been stored (deep freeze) for an unspecified period of time prior to being sold and, as such, the iodine content will not necessarily reflect the time period when the sample was purchased and may even be reduced during the thawing period [54]. Irrespective of any intra- or inter-species variability, the current study is representative of the various seafood iodine contents available to UK consumers.

In the present study, all samples were purchased direct from retailers and fishmongers in the same way that consumers purchase seafood. For many larger species, portions rather than whole fish or whole-side fillets are typically sold. This could perhaps present a complication as levels of other nutrients such as lipids are known to vary throughout the fillet of certain fish species [55,56]. However, Karl et al. [52] found no difference in iodine content when the left and right fillets, as well as dorsal and ventral or head and tail portions from cod were examined. Instead, the same authors observed a higher iodine content, up to 20-fold, in skin as compared to fish muscle with iodine concentrations decreasing from the skin towards the inner part of the fillet closest to the backbone. Accordingly, the dark muscle, which is generally sited close to the skin, has been shown to contain higher iodine contents than the white muscle [54]. All samples in the current study were skinned prior to analysis, as not everyone consumes the skin and some/many portions/fillets are sold skinless. Nonetheless, muscle from the entire portion, including red muscle, was taken and blended to ensure a homogenous sample. Thus, although samples may be potentially lower in iodine than had the skin been left on, they are comparable as the sample preparation was the same for all samples analysed.

In terms of seafood groups, generally higher iodine contents were found in marine species compared to freshwater species, corroborating observations from previous studies [16,38,39,40,57,58]. Using the geometric mean, to account for the skewness of data between the different species within each seafood group, iodine levels were in the order of shellfish (39.3 µg·100 g^−1^) > marine fish (19.8 µg·100 g^−1^) > freshwater fish (6.4 µg·100 g^−1^) (Table 2). Fish obtain iodine through gill and intestinal uptake [3]. The iodine concentration of freshwater is typically much lower than seawater and, as such, the iodine contents of fish largely reflect that of the water they occupy [39,57]. Correspondingly, the two lowest ranking species, common carp and Nile tilapia (*Oreochromis niloticus*), were both freshwater fish. Of the marine fish, the top 8 species were all whitefish belonging to the Gadiformes (e.g., haddock, cod). Only the two hake species, European hake (*Merluccius merluccius*) and Cape hake (*M. capensis*), failed to replicate the high iodine contents measured in the other Gadiformes (range 54.9–427.4 µg·100 g^−1^, ling (Molva molva) to haddock, respectively) containing levels of 13.8 ± 8.0 and 9.7 ± 4.9 µg·100 g^−1^, respectively, which were similar to values reported elsewhere [59,60]. Whitefish, and lean fish in general, have generally been reported as having higher iodine levels than oily fish [15,37,40,51,58]. While lean fish tended to contain higher iodine contents than oily species, no overall correlation between lipid and iodine content per se was observed (*r*^2^ = 0.0033, data not shown) in the present study. This could be related to the wide range and type of samples analysed in the current study with data possibly confounded by the inclusion of farmed, wild, freshwater, marine fish and shellfish. However, even when data were separated to focus solely on wild marine fish species, no correlation was observed (*r*^2^ = 0.0019, data not shown). Furthermore, the precise reasons why iodine contents can vary between fish species are unclear, although they could be related to differences in prey organisms (dietary intake) and/or endogenous metabolism. Separating the shellfish into sub-groups revealed that crustaceans (60.3 µg·100 g^−1^) contained the highest overall iodine contents of any group. Of the molluscs (31.9 µg·100 g^−1^), bivalves (48.3 µg·100 g^−1^) contributed more iodine than cephalopods (8.9 µg·100 g^−1^). Although the vast majority of samples were analysed on a raw basis many of the shellfish products were sold pre-cooked. Blue mussels (Mytilus edulis) were the only species where both a raw and cooked product was tested, with iodine contents higher in cooked (157.6 ± 86.6 µg·100 g^−1^) than raw (104.8 ± 43.9 µg·100 g^−1^). Although the origin of the mussels were different (i.e., raw–farmed, cooked–wild), which itself may result in variation, the result was as expected since, while cooking processes have only minor effects on iodine loss in fish products, high moisture losses during cooking result in increased iodine concentrations on a per weight basis [54]. 

With respect to seafood origin, wild seafood generally contained higher iodine contents than farmed seafood, with exception of non-fed aquaculture species such as mussels and oysters (Figure 1 and Table 3). Farmed fish and prawns are typically fed diets formulated to meet their nutritional needs [61], whereas cultivated bivalves obtain their nutrients through the water in the form of plankton, diatoms and other particulate matter. Thus, the composition of bivalve species such as oysters, mussels and scallops is largely dependent upon the availability of natural food that is available to them, which will vary with location [62]. Nonetheless, comparisons between wild and farmed seafood should be restricted to the same species or their closest counterparts.

### 3.2. Wild Versus Farmed Seafood

**Figure 2:**
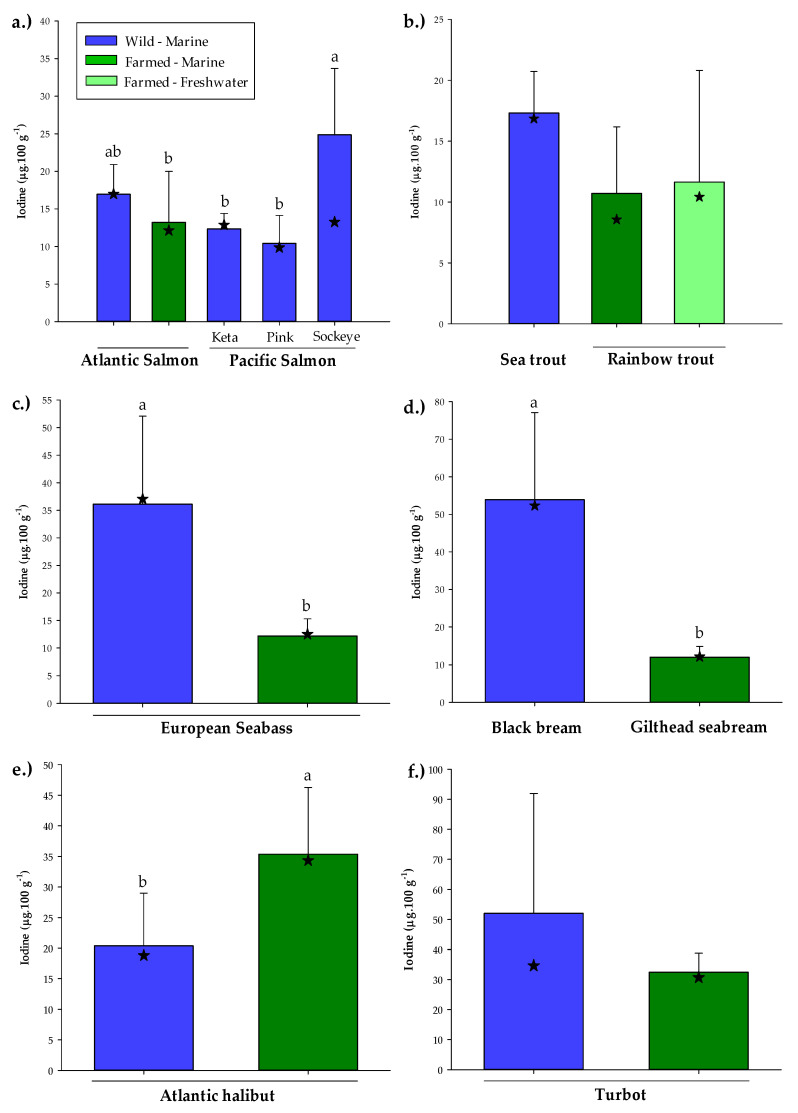
Comparison of iodine contents (µg·100 g^−1^ flesh, ww, mean ± SD) between wild fish and their respective farmed counterparts; (**a**) Atlantic and Pacific salmon, (**b**) sea trout and rainbow trout, (**c**) European seabass, (**d**) black and gilthead seabream, (**e**) Atlantic halibut, (**f**) turbot. Bars bearing different lettering within same species graphs indicate a significant difference (*p* < 0.05). 🟊 indicates median iodine value. Note that scaling for iodine contents differs between species graphs.

In recent years, a greater proportion of seafood destined for the human table market has been supplied by aquaculture, exceeding that provided by wild capture fisheries [30]. Nevertheless, farmed seafood has regularly come under criticism and is often perceived as being an inferior product, in terms of nutritional quality, compared to its wild variant. Of the 95 seafood samples analysed in the current study, 21 were of farmed origin. Of these, eight were identified as having a same-species, or equivalent, wild counterpart. This included the premium food fish salmon, both wild and farmed Atlantic salmon as well as the wild Pacific salmon varieties, keta (*Oncorhynchus keta),* pink (*O. gorbuscha*) and sockeye. Based on the measured iodine contents, no difference was found between wild and farmed Atlantic salmon, 17.0 ± 4.0 and 13.2 ± 6.8 µg·100 g^−1^ flesh ww, respectively (Figure 2a). There was, however, a difference between Atlantic and Pacific species of salmon with sockeye found to contain a significantly higher amount of iodine (24.9 ± 13.3 µg·100 g^−1^) than both farmed Atlantic salmon and the other wild Pacific salmon, keta and pink (12.3 ± 2.1 and 10.4 ± 3.7 µg·100 g^−1^, respectively). Conversely, the mean iodine content for sockeye salmon (13.3 µg·100 g^−1^) was observed to be in the range of all other salmon (9.9–17.0 µg·100 g^−1^). As previously discussed, there are several factors that may affect the iodine content within the same species of wild fish including season and location [38,40]. For the other farmed salmonid, rainbow trout (*O. mykiss*), no significant differences were observed between marine and freshwater reared trout (10.7 ± 5.5 and 11.6 ± 9.2 µg·100 g^−1^), with neither showing differences with wild sea trout (*S. trutta*, 17.3 ± 3.4 µg·100 g^−1^) (Figure 2b). The lack of any difference between the iodine contents of the freshwater and marine reared trout, where respective harvest weights are typically 400 g and 3+ kg, may be somewhat surprising since marine waters, and the organisms that inhabit them, are commonly regarded as being richer sources of iodine [39,57]. Farmed fish, however, are generally supplied with diets that are formulated to at least meet the nutritional requirements of the species being cultured [61]. Thus, supplementation of the farmed feed, either directly or through ingredients used, may negate any expected differences between freshwater and marine-reared fish of the same species, particularly if the dietary iodine level greatly exceed the environmental level. Similarly, any variation in the nutrient composition between individuals of the same farmed species are more likely due to differences in feed formulations brought about by different farming strategies [63].

All of the farmed salmonids, i.e., Atlantic salmon and rainbow trout, irrespective of marine or freshwater culture, contained a similar mean flesh iodine content suggesting that they were fed diets of comparable ingredient compositions. Correspondingly, both farmed seabass and seabream also contained a similar iodine level, ~12.0 µg·100 g^−1^, to the farmed salmonids (Figure 2). However, this was significantly lower than that found in wild seabass (Figure 2c) and seabream (Figure 2d), 36.1 ± 16.0 and 53.9 ± 23.2 µg·100 g^−1^, respectively. One major paradox associated with the increase in aquaculture production to feed a growing population has been the sourcing of suitable ingredients to feed the fish themselves. Farmed fish feeds, particularly for marine carnivorous fish, have shifted from a diet high in the finite and limited marine ingredients, fish meal and fish oil, to a diet high in terrestrial ingredients of plant origin [32]. Consequently, the levels of some of the beneficial nutrients associated with marine ingredient inclusion including long-chain omega-3 fatty acids, selenium and iodine have declined in the feeds and flesh of farmed fish as the use of plant-based ingredients has increased [33,34,35]. As with selenium, the iodine concentrations of plant meals are dependent upon the iodine content and availability of the soils where the crops are cultivated [64,65]. Moreover, cruciferous plants such as rapeseed, which is widely used in aquafeeds [32,35], possess glucosinolates that are known to exert goitrogenic effects through interference of iodine availability and morphological and physiological changes to the thyroid together with reducing palatability and overall growth and production [2,61]. Thus, as iodine is important for fish development [2,3], it may be necessary to supplement feeds containing plant ingredients with greater levels of iodine.

The iodine requirement of farmed fish is estimated to vary between 0.6 and 5.0 mg.kg^−1^ diet, based on rearing conditions, species and life-stage [2,3,61,66]. Freshwater fish for example are more dependent upon dietary sources of iodine. Similarly, fish raised in closed-water systems (i.e., recirculation), as well as systems using ozonised water, are more susceptible to exhibiting signs of goitre, with carnivorous fish affected more than herbivorous and omnivorous fish species [66,67]. This would probably account for the low levels of iodine found in the farmed freshwater, omnivorous carp, tilapia and striped catfish (*Pangasius hypophthalamus*, also known as Basa or river cobbler) of 2.97 ± 1.58, 4.71 ± 1.90 and 6.16 ± 3.46 µg·100 g^−1^ flesh ww, respectively (Figure 1). However, supplementing fish feeds with iodine or iodine-rich sources such as seaweed has been shown to increase fillet levels while also enhancing growth, reducing stress and protecting from disease without affecting thyroid status [2,68,69]. Nevertheless, the iodine contents of aquafeeds at present are most likely sufficient in terms of satisfying the essential requirements of the fish itself, but have resulted in a decline in the amount available to human consumers of the fish.

Atlantic halibut was the only species where the farmed variety was found to contain a significantly higher iodine content than its wild counterpart, 33.4 ± 10.9 and 20.4 ± 8.6 µg·100 g^−1^, respectively (Figure 2e). This is in contrast to Nerhus et al. [40] who reported similar average iodine contents to the present study for wild Atlantic halibut caught from the Barents and Norwegian Seas, 18 and 23 µg·100 g^−1^, respectively, whereas farmed Atlantic halibut was found to contain a lower content of just 11 µg·100 g^−1^. The reasons for the marked differences in iodine contents for farmed halibut between studies may be related to differences in the ingredients used within aquafeeds. The types and inclusion levels of feed ingredients must be formulated to meet the nutrient requirements of the fish [61]. While fish meals are known to generally contain much higher levels of iodine than animal and plant proteins, they are also prone to marked variations in iodine content depending on source. For instance, herring and capelin meals were reported to contain 5–10 mg.kg^−1^, while Atlantic white fish meals can contain upwards of 60–90 mg.kg^−1^ [66]. Additionally, the iodine content of farmed halibut was almost identical to that measured in farmed turbot (32.4 ± 6.4 µg·100 g^−1^), which in turn was lower, but not significantly, than the content of wild turbot (52.0 ± 39.9 µg·100 g^−1^) (Figure 2f). This could indicate that the diets for both farmed halibut and turbot were formulated similarly with respect to both ingredient type and inclusion level, and would further suggest a higher inclusion of marine ingredients than used in the feeds for farmed salmon, trout, seabream and seabass that all exhibited similar iodine contents. European production (including Norway and UK) of farmed halibut (1918 metric tons (MT)) and turbot (10,116 MT) is considerably lower than that of farmed salmon (1,552,335 MT), trout (242,000 MT), seabream (92,000 MT) and seabass (84,400 MT) [70,71]. Thus, both the farmed halibut and turbot sectors can afford to use more of the finite marine ingredients, both in terms of price and volume, in comparison to the species with much larger production. This could also explain why the other farmed salmonid, Arctic char (*Salvelinus alpinus*), which was freshwater-reared and, therefore, would be expected to contain low iodine levels, had a higher content (18.7 ± 6.3 µg·100 g^−1^) than farmed salmon, trout, seabass and seabream, with UK char production approximately 7 MT compared to 166,000 MT for UK farmed salmon [70,72].

It should be noted that certain stocks of wild fish are considered as being critical. Indeed, many of the wild fish species analysed in the present study, such as wild Atlantic salmon and halibut are not commonly found, if at all, in the main fish retailers in the UK (i.e., supermarkets) and are becoming increasingly difficult to source from specialist retailers and fishmongers, in addition to the normal complexities associated with the procurement of wild fish owing to seasonal availability. Farmed fish therefore represent an increasingly important food source in delivering essential nutrients to the human consumer.

### 3.3. Seafood Contribution to Human Iodine Intake

The importance of seafood consumption to human health is globally recognised through dietary guidelines advising routine intake as part of a balanced diet [26,27,28,29]. In the UK, government advice is to consume at least two portions of fish per week, of which at least one should be an oily fish, with serving size defined as 140 g [28]. Based on these expectations, a single serving of the highest iodine species from the present study, the whitefish Atlantic haddock, would provide 598 µg or 427% of the UK’s 140 µg·day^−1^ RNI for adults [6], equivalent to 61.1% of a 980 µg weekly recommended iodine intake (see Table S1). Conversely, a 140 g serving of the highest iodine containing ‘oily’ fish species, Atlantic mackerel, would supply just 4.6% (48.1 µg) of the total recommended weekly intake (34.4% of daily intake), whereas the lowest species overall, freshwater farmed common carp serves just 0.42% (4.2 µg or 3.0% of daily intake). Oily fish are particularly recommended in the human diet due to their high contents of omega-3 long-chain polyunsaturated fatty acids, namely eicosapentaenoate and docosahexaenoate, and their molecular and cellular effects in supporting optimal cell and tissue function together with promoting health [73]. Although emphasis has predominantly been placed on fish consumption in dietary guidelines, it is also important to recognise the contribution from other seafood sources. In terms of iodine intake for example, a serving of brown crab meat, which also has a relatively high lipid content (~11 g·100 g^−1^), would provide 32.2% (315.2 µg), and langoustines (*Nephrops norvegicus*) 48.8% (478.4 µg) of the weekly recommendation. Nevertheless, eating one portion of both haddock and mackerel per week would contribute approximately two-thirds of the recommended weekly iodine intake for UK adults. The remainder, therefore, would be expected to be satisfied through other dietary sources with milk and dairy products being the main sources of iodine intake in the UK [6,16], estimated to provide one-third of an adult’s RNI [6]. Thus, by adhering to current seafood intake recommendations, and selecting species high in iodine, UK consumers can effectively meet the RNI when assessed over the course of a week.

**Figure 3:**
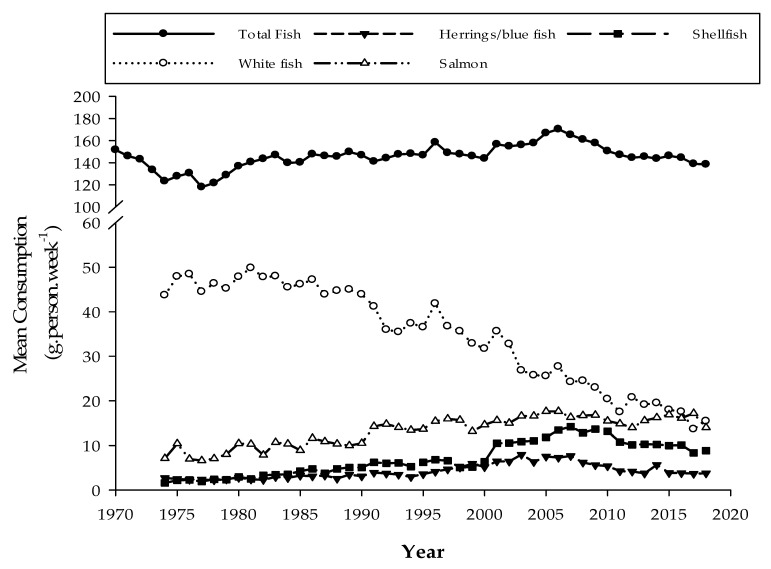
Mean UK consumption (g·person·week^−1^) of total fish (^__^●^__^) and breakdown of major subgroups: whitefish (^….^○^….^), herring/blue fish (^_ _^ ▼ ^_ _^), salmon (^_.._^∆^.._..^), and shellfish (^__ _^■^_ __^). Data extrapolated from the UK’s Family Food datasets 2018/19 [74].

The above assumptions are, nonetheless, dependent upon the UK population following guidelines and consuming at least two 140 g servings of fish/seafood per week (i.e., 280 g total). However, data from the UK’s annual national ‘Family Food’ survey show that the mean UK consumption of seafood currently stands at approximately half the recommended level at 138.5 g·person·week^−1^ [74] (Figure 3). Moreover, consumption of whitefish, which the present study has demonstrated are rich sources of iodine, has fallen significantly since 1974 from 44 g·person·week^−1^ to a current intake level of approximately 15 g·person·week^−1^, although this excludes whitefish included within takeaway and processed ready meals (6.9 g·person·week^−1^). Oily fish consumption (herring/blue fish, etc.) has remained constant approximately 3 g·person·week^−1^, whereas salmon consumption doubled over the same period from 7 to 14 g·person·week^−1^, reflecting the period when intensive farmed salmon production commenced ensuring its dominance within UK and European markets as one of the most consumed fish species [75,76]. Over time, there has been a gradual shift by consumers towards purchasing more farmed species of fish than traditional wild caught species such that they now account for 38.2% by volume of the UK top five bestselling seafood species [76]. Similarly, shellfish consumption has increased from ~2 to 9 g person.week^−1^. These values are based on a UK average but not everyone eats seafood, either as a result of dietary and/or lifestyle choices (vegan, vegetarian) or through personal choice due to price, difficulty preparing/cooking, or dislike of smell [77,78].

Data from the UK’s National Diet and Nutrition Survey (NDNS), based on consumption patterns over a 4 day dietary recording period, indicate that approximately 60% of those aged 19–64 consume fish, rising to 80% for those aged 65 and over [79], a trend that has been observed in other countries [11,59]. In children, 70% of those aged 1.5–10 years are estimated to consume fish, whereas only 50% of 11–18 year olds are consumers with males (~55%) consuming more than their female counterparts (~45%). It is this latter cohort of women entering into reproductive age together with pregnant individuals that are generally identified as being more at risk of iodine deficiencies owing to the problems it poses to foetal and neonatal development [4,5,11,12,13,14]. However, calculation of seafood intakes from NDNS disaggregated seafood data (years 1–8 combined) indicates that this age group (11–18) consumes the most fish of all UK age groups (36.3 g·day^−1^), equivalent to 90.8% of the minimum 280 g recommended intake based on two servings of 140 g [28], although females still consume less than their male counterparts (34.2 and 38.5 g·day^−1^ or 85.5 and 96.3%, respectively) (Table 4). High fish consumption has been estimated to result in higher iodine intake compared to low consumers, based on validated food frequency questionnaire [77], although the type of fish consumed will invariably affect the overall iodine intake. Applying the iodine results from the present study to the NDNS consumption data by both age and gender shows that seafood contributes between 9.4% (13.1 µg·day^−1^) of the daily RNI for iodine in females aged 19–64 and 18.0% (12.6 µg·day^−1^) in males aged 1–5 years (Table 5), where UK RNIs range from 70 µg·day^−1^ for 1–3 year olds to 140 µg·day^−1^ for those aged 15+ [80]. As expected, whitefish is the main contributor to iodine intake across all age groups and genders, accounting for approximately 60–70% of the total seafood iodine intake, with the exception of the youngest age group (1–5 years) where ‘other’ seafood contributed an equal or slightly higher intake (Table 5). This ‘other’ seafood group includes processed fish products, including ready meals, such as fish fingers which are typically comprised of whitefish species and, therefore, a rich source of iodine and tend to be a popular meal choice among infants and young children. The contribution of the remaining seafood groups, i.e., salmon, shellfish and tuna, was generally quite low across all age and gender classes (1.4–8.8% of total iodine intake) due to a combination of low iodine contents and/or low consumption levels. These data, however, include both consumers and non-consumers of seafood. Thus, actual contribution to iodine intake for just seafood consumers would be higher. For example, given that approximately 50% of females aged 11–18 eat seafood [79], one can assume that the actual contribution of seafood to iodine intake in consumers is approximately 34 µg·day^−1^, equivalent to 24–26% of the RNI. This is similar to adolescent Icelandic girls where fish was estimated to provide 24% of total dietary intake and where 94% of the subjects consumed fish [81]. Still, the contribution of seafood to total iodine intake in the UK across all age groups and genders is relatively low given its importance, with females obtaining less than their same age male counterparts. The overall value for money (quality/taste and health) rather than price *per se* is the main factor highlighted affecting UK seafood consumption, despite costing four times the price of meat on average [76]. It is worth highlighting that the use of 4 day dietary records may not be suitable for use with food groups that are consumed less frequently, such as the consumption of seafood where at least two portions of fish per week are recommended, since there is the potential for missing days when seafood is consumed. Alternative methods to assessing iodine status include measuring the urinary iodine concentration (UIC) as the majority of iodine absorbed by the body is excreted via the urine, although this typically reflects iodine intake within the last 48 h [82]. Øyen et al. [83] recently demonstrated a direct link between seafood intake and UIC through a 4 week lean seafood intervention study, resulting in an increased dietary iodine concentration from baseline levels as compared to non-seafood consuming participants. Routine seafood consumption therefore has the capacity to maintain a high level of intake in order to help prevent iodine deficiency disorders, although making this habit appeal to younger consumers still remains a challenge.

## 4. Conclusions

Data on the iodine contents of food are important in order to maintain food composition databases and estimate an individual’s intake status. The present study provides iodine data on a comprehensive list of seafood species available to the UK consumer, extending our knowledge from the handful of species commonly consumed to those growing in popularity while highlighting the large variations in iodine contents that can occur. Shellfish generally provide the highest contents of iodine as compared to marine and freshwater fish, respectively, with crustaceans, whitefish (Gadiformes) and bivalves contributing the most. Although wild fish tend to contain higher iodine contents than farmed species, few differences were observed when comparing equivalent wild-farmed species, with variations most likely explained by ingredient type and levels used in feeds for farmed fish. Overall, seafood consumption has the means to provide approximately two-thirds of the UK recommended intake when following the recommended dietary intake of one portion of oily and one of lean fish species per week although, in reality, current seafood intake provides just 10–20% of the UK RNI for iodine, with males achieving higher levels than their female counterparts.

## Figures and Tables

**Table 1:** List of the 95 seafood samples analysed for iodine content in the current study. All samples raw unless indicated by *.

Common Name ^1^	Scientific Name	Farmed/Wild	Location ^2^	*N*
*FRESHWATER FISH*				
Arctic char	*Salvelinus alpinus*	Farmed	UK	4
Common carp	*Cyprinus carpio*	Farmed	Poland	5
Milkfish	*Chanos chanos*	Farmed	Indonesia	4
Nile tilapia	*Oreochromis niloticus*	Farmed	China	4
Rainbow trout	*Oncorhynchus mykiss*	Farmed	UK	5
Siberian sturgeon	*Acipenser baerii*	Farmed	France	4
Striped catfish (Basa Pangasius)	*Pangasius hypophthalmus*	Farmed	Vietnam	5
Zander (pike-perch)	*Sander lucioperca*	Wild	Kazakhstan	4
***MARINE FISH***				
***Anguilliformes***				
Daggertooth pike-conger	*Muraenesx cinerus*	Wild	NE Atlantic (FAO 27)	4
European conger	*Conger conger*	Wild	NE Atlantic (FAO 27)	4
***Clupeiformes***				
Atlantic herring	*Clupea harengus*	Wild	NE Atlantic (FAO 27 IV, VII)	7
European anchovy	*Engraulis encrasicolus*	Wild	W. Mediterranean (FAO 37.1)	3
European pilchard (sardine)	*Sardina pilchardus*	Wild	NE Atlantic (FAO 27 IV, VII)	5
European sprat	*Sprattus sprattus*	Wild	NE Atlantic (FAO 27 VII)	6
***Gadiformes***				
Atlantic cod	*Gadus morhua*	Wild	NE Atlantic (FAO 27 I, II, IV, V)	6
Alaskan pollock	*Theragra chalcogramma*	Wild	NE Pacific (FAO 67) NW Pacific (FAO 61)	3
Cape hake	*Merluccius capensis*	Wild	SE Atlantic (FAO 47)	4
European hake	*Merluccius merluccius*	Wild	NE Atlantic (FAO 27 IV, VII)	4
Haddock	*Melanogrammus aeglefinus*	Wild	NE Atlantic (FAO 27 I, II, IV, V, VII)	5
Ling	*Molva molva*	Wild	NE Atlantic (FAO 27)	5
Pollack (Atlantic pollock)	*Pollachius pollachius*	Wild	NE Atlantic (FAO 27 IV)	3
Pouting	*Trisopterus luscus*	Wild	NE Atlantic (FAO 27 IV, VII)	3
Saithe (Coley)	*Pollachius virens*	Wild	NE Atlantic (FAO 27 IV, VII)	4
Whiting	*Merlangius merlangus*	Wild	NE Atlantic (FAO 27 IV, VII)	5
***Lophiformes***				
Monkfish	*Lophius piscatorius*	Wild	N.E. Atlantic (FAO 27)	4
***Mugiliformes***				
Flathead grey mullet	*Mugil cephalus*	Wild	N.E. Atlantic (FAO 27)	5
Thicklip grey mullet	*Chelon labrosus*	Wild	N.E. Atlantic (FAO 27)	4
***Percoideri***				
Atlantic horse mackerel	*Trachurus trachurus*	Wild	NE Atlantic (FAO 27 IX)	3
Barramundi (Asian seabass)	*Lates calcarifer*	Farmed	Vietnam	6
Black seabream	*Spondyliosoma cantharus*	Wild	NE Atlantic (FAO 27)	6
European seabass	*Dicentrachus labrax*	Wild	NE Atlantic (FAO 27)	4
		Farmed	Greece, Turkey	6
Gilthead seabream	*Sparus aurata*	Farmed	Greece, Turkey	6
Meagre	*Argyrosomus regius*	Farmed	Greece, Turkey	4
Red mullet (Indian goatfish)	*Parupeneus indicus, P. heptacanthus*	Wild	W Indian (FAO 51), E Indian (FAO 57)	8
Red snapper	*Lutjanus malabaricus, L. sebae, Pinjalo pinjalo*	Wild	W Central Pacific (FAO 71)	4
White trevally (Trevally jack)	*Pseudocaranx dentex*	Wild	SW Pacific (FAO 81)	3
Yellow croaker	*Larimichthys polyactis*	Wild	NW Pacific (FAO 61)	3
***Pleuronectiformes***				
Atlantic halibut	*Hippoglossus hipposglossus*	Wild	NE Atlantic (FAO 27)	6
		Farmed	Norway	6
Brill	*Scophthalmus rhombus*	Wild	NE Atlantic (FAO 27)	5
Common dab	*Limanda limanda*	Wild	NE Atlantic (FAO 27 IV, VII)	4
Common sole (Dover sole)	*Solea solea*	Wild	NE Atlantic (FAO 27 IV, VII)	6
European flounder	*Platichys flesus*	Wild	NE Atlantic (FAO 27)	4
European plaice	*Pleuronectes platessa*	Wild	NE Atlantic (FAO 27 IV, V, VII)	6
Lemon sole	*Microstomus kitt*	Wild	NE Atlantic (FAO 27 IV, V, VII)	7
Megrim	*Lepidorhombus whiffiagonis*	Wild	NE Atlantic (FAO 27 IV, VII)	4
Turbot	*Psetta maxima (Scophthalmus maximus)*	Wild	NE Atlantic (FAO 27)	5
Witch flounder (Witch sole)	*Glyptocephalus cynoglossus*	Wild	NE Atlantic (FAO 27)	5
Yellowfin sole	*Limanda aspera*	Wild	NE Pacific Ocean (FAO 67)	3
***MARINE FISH***				
***Rajiformes***				
Ray wings	*Leucoraja naevus, Raja montagui, R. clavata*	Wild	NE Atlantic (FAO 27 II, IV, VI, VII)	4
***Salmoniformes***				
Atlantic salmon	*Salmo salar*	Wild	UK, Norway	6
		Farmed	UK, Norway	32
Keta salmon (Chum)	*Oncorhynchus keta*	Wild	NE Pacific (FAO 67)	5
Pink salmon (Humpback)	*Oncorhynchus gorbuscha*	Wild	NE Pacific (FAO 67)	4
Rainbow trout	*Oncorhynchus mykiss*	Farmed	UK	5
Sea trout	*Salmo trutta*	Wild	NE Atlantic (FAO 27 IV)	4
Sockeye salmon (Red)	*Oncorhynchus nerka*	Wild	NE Pacific (FAO 67), NW Pacific (FAO 61)	6
***Scombroidei***				
Albacore tuna	*Thunnus alalunga*	Wild	W Indian (FAO 51) E Indian (FAO 57), W Central Pacific (FAO 71) E Central Pacific (FAO 77) W Central Atlantic (FAO 31)	5
Atlantic mackerel	*Scomber scombrus*	Wild	NE Atlantic (FAO 27 IV, VI, VII, VIII)	7
Black marlin	*Makaira indica*	Wild	W Central Pacific (FAO 71)	5
Largehead hairtail (Beltfish)	*Trichiurus lepturus*	Wild	W Central Pacific (FAO 71)	3
Savalai hairtail (Ribbonfish)	*Lepturacanthus savala*	Wild	W Indian (FAO 51)	3
Swordfish	*Xiphias gladius*	Wild	E Indian (FAO 57) W Indian (FAO 51) E Central Pacific (FAO 77) W Central Pacific (FAO 71)	5
Wahoo (Kingfish)	*Acanthocybium solandri*	Wild	E Indian (FAO 57) W Indian (FAO 51)	4
Yellowfin tuna	*Thunnus albacares*	Wild	E Indian (FAO 57) W Indian (FAO 51) W Central Pacific (FAO 71) E Central Pacific (FAO 77) SE Pacific (FAO 87) SW Pacific (FAO 81)	6
***Scorpaeniformes***				
Gurnard	*Chelidonichthys lucerna, C. spinosus, Eutrigla gurnadus*	Wild	NE Atlantic (FAO 27 IV, VII)	8
Redfish (Norway Redfish)	*Sebastes* spp.	Wild	NE Atlantic (FAO 27 V)	4
***Stromateoidei*, *Anabantoidei***				
Silver pomfret	*Pampus argentus*	Wild	W Indian (FAO 51)	4
***Squaliformes***				
Picked dogfish (Huss)	*Squalus acanthias*	Wild	NW Atlantic (FAO 21)	3
***Zeiformes***				
John dory	*Zeus faber*	Wild	NE Atlantic (FAO 27)	7
Oreo dory (Smooth oreo)	*Pseudocyttus maculatus*	Wild	SW Pacific (FAO 81)	5
***Zoarcoidei***				
Atlantic wolfish	*Anarhichas lupus*	Wild	NE Atlantic (FAO 27)	4
***Scombroidei***				
Albacore tuna	*Thunnus alalunga*	Wild	W Indian (FAO 51) E Indian (FAO 57), W Central Pacific (FAO 71) E Central Pacific (FAO 77) W Central Atlantic (FAO 31)	5
Atlantic mackerel	*Scomber scombrus*	Wild	NE Atlantic (FAO 27 IV, VI, VII, VIII)	7
Black marlin	*Makaira indica*	Wild	W Central Pacific (FAO 71)	5
***SHELLFISH***				
***CRUSTACEANS***				
American lobster	*Homarus americanus*	Wild	NW Atlantic (FAO 21)	4
* Edible crab—whole	*Cancer pagrus*	Wild	NE Atlantic (FAO 27 VII)	3
* Edible crab—brown meat		Wild	NE Atlantic (FAO 27 IV, VII)	4
* Edible crab—white meat		Wild	NE Atlantic (FAO 27 VII)	3
King prawn	*Litopenaeus vannamei*	Farmed	Vietnam, Ecuador, Honduras	4
Langoustines (Norway lobster)	*Nephrops norvegicus*	Wild	NE Atlantic (FAO 27)	4
Tiger Prawn	*Penaeus monodon*	Farmed	Vietnam	4
***MOLLUSCS***				
***Bivalves***				
Atlantic (deep-sea) scallop	*Placopecten magellanicus*	Wild	NW Atlantic (FAO 21)	3
Atlantic razor clam	*Ensis directus*	Wild	NE Atlantic (FAO 27 IV??)	5
* Blue mussels	*Mytilus edulis*	Wild	NE Atlantic (FAO 27)	4
Blue mussels		Farmed	UK	4
* Chilean mussels	*Mytilus chilensis*	Farmed	Chile	4
***SHELLFISH***				
***MOLLUSCS***				
***Bivalves.***				
* Common edible cockle	*Cerastoderma edule*	Wild	NE Atlantic (FAO 27 IV)	4
Green-lipped mussels	*Perna canaliculus*	Farmed	New Zealand	4
King scallop + roe	*Pecten maximus*	Wild	NE Atlantic (FAO 27)	3
Pacific oyster	*Crassostrea gigas*	Farmed	UK, Ireland	3
Patagonian scallop	*Zygochlamys patagonica*	Wild	SW Atlantic (FAO 41)	3
***Cephalopods***				
Pharaoh cuttlefish	*Sepia pharaonis*	Wild	W Indian (FAO 51)	4
Horned octopus (curled)	*Eledone cirrhosa*	Wild	NE Atlantic (FAO 27 IV, VII)	5
Squid	*Loligo vulgaris*, *L. forbesi*	Wild	W Central Pacific (FAO 71) E Indian (FAO 57) W Indian (Area 51)	4

^1^ FAO nomenclature [47]. ^2^ Location according to product label/information provided and relevant FAO fishing ground [48], where known. * All samples raw unless indicated by *.

**Table 2:** Iodine content of seafood (µg·100 g^−1^ flesh ww) based on the different seafood classifications.

Common Name ^1^	*n*	Iodine Content (µg·100 g^−1^ Flesh ww)				
		Mean	Geo-Mean	Median	Min.	Max.
**FRESHWATER FISH**	**35**	**8.27**	**6.52**	**6.40**	**1.13**	**26.46**
**MARINE FISH**	**342**	**31.17**	**19.97**	**17.31**	**3.49**	**909.77**
*Anguilliformes*	*8*	*21.90*	*20.79*	*20.44*	*13.60*	*41.53*
*Clupeiformes*	*21*	*26.55*	*23.59*	*23.72*	*10.83*	*69.70*
*Gadiformes*	*42*	*100.72*	*54.69*	*65.14*	*5.97*	*909.77*
*Lophiformes*	*4*	*33.35*	*31.90*	*33.56*	*21.95*	*41.44*
*Mugiliformes*	*9*	*35.08*	*18.29*	*13.81*	*7.18*	*197.26*
*Percoideri*	*53*	*21.48*	*17.70*	*16.11*	*5.94*	*95.78*
*Pleuronectiformes*	*66*	*27.06*	*22.91*	*23.48*	*7.07*	*103.01*
*Rajiformes*	*4*	*16.63*	*16.11*	*16.62*	*10.88*	*22.42*
*Salmoniformes*	*62*	*14.51*	*13.14*	*13.64*	*4.54*	*34.39*
*Scombroidei*	*38*	*17.48*	*14.43*	*13.82*	*6.04*	*58.50*
*Scorpaeniformes*	*12*	*24.07*	*20.52*	*19.41*	*8.42*	*66.43*
*Stromateoidei*, *Anabantoidei*	*4*	*5.35*	*5.28*	*5.40*	*4.12*	*6.48*
*Squaliformes*	*3*	*26.29*	*25.25*	*30.45*	*16.65*	*31.76*
*Zeiformes*	*12*	*12.52*	*10.43*	*13.61*	*3.49*	*27.81*
*Zoarcoidei*	*4*	*38.34*	*28.78*	*36.95*	*12.75*	*66.71*
**SHELLFISH**	**79**	**86.09**	**39.33**	**58.41**	**3.81**	**440.18**
**Crustaceans**	**26**	**139.98**	**60.26**	**96.36**	**3.81**	**440.18**
**Molluscs**	**53**	**59.66**	**31.90**	**49.37**	**3.92**	**241.45**
*Bivalves*	*40*	*75.71*	*48.29*	*77.77*	*5.59*	*241.45*
*Cephalopods*	*13*	*10.28*	*8.90*	*6.62*	*3.92*	*19.97*

^1^ Refer to Table 1 for full breakdown of species within each seafood class.

**Table 3:** Iodine contents (µg·100 g^−1^ flesh ww) of wild and farmed seafood in the major seafood groups.

Common Name ^1^	*n*	Iodine Content (µg·100 g^−1^ Flesh ww)				
		Mean	Geo-Mean	Median	Min.	Max.
**WILD (All)**	**332**	**44.89**	**24.07**	**19.55**	**3.49**	**909.77**
**FARMED (All)**	**124**	**22.97**	**13.61**	**11.72**	**1.13**	**169.64**
**FRESHWATER FISH**						
*Wild*	*4*	*5.77*	*5.70*	*5.43*	*5.03*	*7.18*
*Farmed*	*31*	*8.59*	*6.63*	*6.84*	*1.13*	*26.46*
**MARINE**						
*Wild (All)*	*328*	*45.36*	*24.49*	*19.70*	*3.49*	*909.77*
*Farmed (All)*	*93*	*27.77*	*17.30*	*14.19*	*3.81*	*169.64*
***MARINE FISH***						
*Wild*	*272*	*35.08*	*22.03*	*18.86*	*3.49*	*909.77*
*Farmed*	*70*	*15.97*	*13.65*	*13.20*	*4.54*	*49.61*
***SHELLFISH***						
*Wild (All)*	*56*	*95.31*	*40.99*	*51.49*	*3.92*	*440.18*
*Farmed (All)*	*23*	*63.66*	*35.55*	*59.13*	*3.81*	*169.64*
***Crustaceans***						
*Wild*	*18*	*199.13*	*162.66*	*197.92*	*41.87*	*440.18*
*Farmed*	*8*	*6.89*	*6.51*	*6.73*	*3.81*	*11.13*
***Molluscs***						
*Wild (All)*	*38*	*46.13*	*21.37*	*16.05*	*3.92*	*241.45*
*Farmed (All)*	*15*	*93.94*	*87.95*	*84.12*	*49.37*	*169.64*
***Bivalves***						
*Wild*	*25*	*64.77*	*33.70*	*43.94*	*5.59*	*241.45*
*Farmed*	*15*	*93.94*	*87.94*	*84.12*	*49.37*	*169.64*
Cephalopods *	***13***	***10.28***	***8.90***	***6.62***	***3.92***	***19.97***

^1^ Refer to Table 1 for full breakdown of species within each seafood class. * Wild species only.

**Table 4:** Seafood intake (g·day^−1^) of UK individuals, based on age and gender, including non-consumers, calculated from NDNS disaggregated data seafood type (years 1–8 combined [50]).

Fish Intake (g·Day^−1^)												
Age Range (Years)	Males and Females				Males				Females			
	1–5	6–10	11–18	19–64	1–5	6–10	11–18	19–64	1–5	6–10	11–18	19–64
*Seafood type*												
Salmon	2.0	2.6	4.4	4.2	1.9	2.8	4.4	4.3	2.0	2.3	4.4	4.2
Other Oily	1.0	1.4	1.5	3.5	1.1	1.5	1.5	3.9	0.8	1.3	1.5	3.2
***Total Oily***	***3.0***	***4.0***	***5.9***	***7.7***	***3.0***	***4.3***	***5.9***	***8.2***	***2.8***	***3.6***	***5.9***	***7.4***
Other	12.0	10.5	6.3	3.3	12.9	11.3	6.9	3.5	10.9	9.5	5.7	3.1
Tuna	3.1	5.1	9.5	4.8	2.7	4.9	9.4	5.4	3.6	5.3	9.6	4.3
Whitefish	4.9	8.2	11.2	8.7	4.7	9.2	13.0	10.6	5.0	7.2	9.6	7.4
***Total Non-Oily***	***20.0***	***23.8***	***27.0***	***16.8***	***20.3***	***25.4***	***29.3***	***19.5***	***19.5***	***22.0***	***24.9***	***14.8***
Shellfish	0.7	1.4	3.3	3.2	0.8	1.8	3.2	3.2	0.6	0.9	3.4	3.3
***TOTAL***	***23.7***	***29.2***	***36.2***	***27.7***	***24.1***	***31.5***	***38.4***	***30.9***	***22.9***	***26.5***	***34.2***	***25.5***
%RWI *	***59.3***	***73.0***	***90.5***	***69.3***	***60.3***	***78.8***	***96.0***	***77.3***	***57.3***	***66.3***	***85.5***	***63.8***

* UK recommended weekly intake (RWI) is 280 g based on two 140 g servings [28]. Bold italics show the sum of the groups included within Oil and Non-Oily sections as well as the overall Total which includes the shellfish group. Bold is also required for the important %RWI.

**Table 5:** Estimated iodine intake (µg·day^−1^) of UK individuals, based on age and gender, including non-consumers, from seafood consumption using iodine results from the present study and seafood intake calculated from NDNS disaggregated data seafood type (years 1–8 combined [50]) shown in Table 4. Data shown include non-seafood consumers.

Estimated Iodine Intake (g·Day^−1^)												
Age Range (Years)	Males and Females				Males				Females			
	1–5	6–10	11–18	19–64	1–5	6–10	11–18	19–64	1–5	6–10	11–18	19–64
*Seafood type*												
Salmon	0.30	0.40	0.64	0.60	0.31	0.42	0.66	0.60	0.30	0.37	0.63	0.59
Other Oily	0.22	0.29	0.30	0.73	0.24	0.35	0.31	0.85	0.21	0.21	0.28	0.64
***Total Oily***	***0.52***	***0.69***	***0.94***	***1.33***	***0.55***	***0.77***	***0.97***	***1.45***	***0.51***	***0.58***	***0.91***	***1.23***
Other	5.67	4.88	2.76	1.27	6.11	5.25	3.23	1.36	5.18	4.46	2.30	1.21
Tuna	0.28	0.46	0.85	0.43	0.25	0.44	0.86	0.49	0.32	0.49	0.84	0.40
Whitefish	5.27	9.33	13.38	10.72	5.05	10.74	15.23	13.00	5.52	7.75	11.62	9.13
***Total Non-Oily***	***11.22***	***14.67***	***16.99***	***12.42***	***11.41***	***16.43***	***19.32***	***14.85***	***11.02***	***12.70***	***14.76***	***10.74***
Shellfish	0.48	0.56	1.23	1.18	0.61	0.65	1.18	1.21	0.33	0.44	1.28	1.16
***TOTAL***	***12.2***	***15.9***	***19.2***	***14.9***	***12.6***	***17.8***	***21.5***	***17.5***	***11.8***	***13.7***	***16.9***	***13.1***
%RNI *	***12.2–17.4***	***14.5–15.9***	***13.7–14.8***	***10.6***	***12.6–18.0***	***16.2–17.8***	***15.4–16.5***	***12.5***	***11.8–16.9***	***12.5–13.7***	***12.1–13.0***	***9.4***

* UK reference nutrient intake (RNI) for iodine: 1–3 years 70 µg·day^−1^, 4–6 years 100 µg·day^−1^, 7–10 years 110 µg·day^−1^, 11–14 years 130 µg·day^−1^, and 15+ years 140 µg·day^−1^ [80]. Bold italics show the sum of the groups included within Oil and Non-Oily sections as well as the overall Total which includes the shellfish group. Bold is also required for the important %RWI.

## Data Availability

Not applicable.

## Supplementary Materials

The following are available online at https://www.mdpi.com/article/10.3390/nu14010195/s1, Table S1: Mean, SD, median and range (min-max) of iodine contents (µg·100 g^−1^ flesh ww) analysed in the 95 seafood samples in the current study and their contribution to a weekly 980 µg recommended intake for iodine in UK adults, based on the 140 µg RNI and a suggested 140 g seafood serving size. All samples analysed raw unless indicated by *. Refer to Table 1 for species scientific name and farmed/wild-catch location.
