# Supplementary material for: Iodine Content of Wild and Farmed Seafood and Its Estimated Contribution to UK Dietary Iodine Intake
Source: Nutrients. 2021 Dec 31;14(1):195. doi: 10.3390/nu14010195 (PMC8747335; doi:10.3390/nu14010195)
Supplement: Supplementary file 1 [file nutrients-14-00195-s001.zip › nutrients-1523346-supplementary-done.pdf]

nutrients

MDPI

Supplementary Table S1. Mean, SD, median and range (min-max) of iodine contents (µg.100 g^{-1} flesh ww) analysed in the 95 seafood samples in the current study and their contribution to a weekly 980 µg recommended intake for iodine in UK adults, based on the 140 µg RNI and a suggested 140 g seafood serving size [6,28]. All samples analysed raw unless indicated by *. Refer to Table 1 for species scientific name and farmed/wild-catch location.

Common name	Wild/ Farmed	Iodine (µg.100 g^{-1} flesh)				% RWI, 140g serving	*n*
		Mean	SD	Median	Range (min-max)		
**FRESHWATER FISH**							
Arctic char	Farmed	18.72	6.33	20.14	10.01-24.59	2.67	4
Common carp	Farmed	2.97	1.58	2.67	1.13-3.34	0.42	5
Milkfish	Farmed	8.50	3.53	7.30	5.83-13.58	1.21	4
Nile tilapia	Farmed	4.71	1.90	4.48	3.00-6.90	0.67	4
Rainbow trout	Farmed	11.64	9.17	10.43	2.99-26.46	1.66	5
Siberian sturgeon	Farmed	8.69	1.32	8.96	6.84-9.99	1.24	4
Striped catfish (Basa, Pangasius)	Farmed	6.16	3.46	4.88	2.95-10.95	0.88	5
Zander (pike-perch)	Wild	5.77	0.96	5.43	5.03-7.18	0.82	4
**MARINE FISH**							
Anguilliformes							
Daggertooth pike conger	Wild	25.16	10.96	20.44	18.25-41.53	3.59	4
European conger	Wild	18.63	4.44	19.08	13.60-22.76	2.66	4
Clupeiformes							
Atlantic herring	Wild	30.41	25.08	16.76	11.90-69.70	4.34	7
European anchovy	Wild	32.73	2.20	31.66	31.26-35.26	4.68	3
European pilchard (sardine)	Wild	24.77	4.45	24.78	18.26-30.45	3.54	5
European sprat	Wild	20.44	6.42	19.52	10.83-29.46	2.92	6
Gadiformes							
Atlantic cod	Wild	70.72	18.96	74.35	40.33-94.26	10.10	6
Alaskan Pollock	Wild	56.83	9.13	53.50	49.83-67.17	8.12	3
Cape hake	Wild	9.67	4.93	7.99	5.97-16.74	1.38	4
European hake	Wild	13.84	7.98	10.31	8.96-25.78	1.98	4
Haddock	Wild	427.38	316.09	323.27	80.77-909.77	61.05	5
Ling	Wild	54.88	33.66	63.11	16.66-87.96	7.84	5
Pollack (Atlantic Pollock)	Wild	76.05	24.65	72.48	53.38-102.29	10.86	3
Pouting	Wild	82.04	26.65	89.11	52.56-104.44	11.72	3
Saithe (Coley)	Wild	85.81	31.19	95.28	42.15-110.53	12.26	4
Whiting	Wild	62.49	24.77	52.83	34.62-99.18	8.93	5
Lophiformes							
Monkfish	Wild	33.35	11.18	33.56	21.95-44.34	4.76	4
Mugiliformes							
Flathead grey mullet	Wild	52.21	81.53	21.89	7.18-197.26	7.46	5
Thicklip grey mullet	Wild	13.66	1.24	13.67	12.14-15.15	1.95	4

Percoidei							
Atlantic horse mackerel	Wild	22.85	2.06	22.12	21.25-25.18	3.26	3
Barramundi (Asian seabass)	Farmed	10.15	3.92	10.27	5.94-12.87	1.45	6
Black seabream	Wild	53.88	23.19	52.32	27.37-95-78	7.70	6
European seabass	Wild	36.11	15.97	37.06	16.42-53.89	5.16	4
	Farmed	12.18	3.10	12.50	8.13-16.11	1.74	6
Gilthead seabream	Farmed	12.03	2.82	12.13	7.23-15.29	1.72	6
Meagre	Farmed	15.33	5.57	17.06	7.56-19.66	2.19	4
Red mullet (Indian goatfish)	Wild	21.76	6.35	19.44	13.71-33.04	3.11	8
Red snapper	Wild	19.04	6.42	18.48	12.52-26.69	2.72	4
White trevally (Trevally jack)	Wild	17.11	1.38	17.59	15.56-18.20	2.44	3
Yellow croaker	Wild	11.11	1.76	10.76	9.55-13.01	1.59	3
Pleuronectiformes							
Atlantic halibut	Wild	20.37	8.61	18.80	11.07-32.23	2.91	7
	Farmed	35.35	10.90	34.33	22.13-49.61	5.05	6
Brill	Wild	21.77	4.80	23.72	15.63-27.52	3.11	5

Supplementary Table S1 Cont.

**Common name**	**Wild/ Farmed**	**Iodine (µg.100 g^{-1} flesh)**				**% RWI, 140g serving**	***n***
		**Mean**	**SD**	**Median**	**Range (min-max)**		
**MARINE FISH Cont.**							
Pleuronectiformes cont.							
Common dab	Wild	16.71	2.94	16.71	13.14-20.28	2.39	4
Common sole (Dover sole)	Wild	25.18	13.37	21.38	13.38-47.26	3.60	7
European flounder	Wild	20.29	11.13	20.36	9.34-31.11	2.90	4
European plaice	Wild	15.52	6.58	13.90	9.83-26.65	2.22	6
Lemon sole	Wild	26.12	26.39	15.54	7.07-84.09	3.73	7
Megrim	Wild	17.71	9.90	17.18	7.75-28.72	2.53	4
Turbot	Wild	52.01	39.87	34.60	13.91-103.01	7.43	5
	Farmed	32.42	6.36	30.65	26.74-43.38	4.63	5
Witch flounder (Witch sole)	Wild	41.38	5.88	41.62	33.39-49.89	5.91	5
Yellowfin sole	Wild	22.55	9.58	20.36	14.25-33.03	3.22	3
Rajiformes							
Ray wings	Wild	16.63	4.72	16.62	10.88-22.42	2.38	4
Salmoniformes							
Atlantic salmon	Wild	16.95	3.97	16.98	11.95-23.21	2.42	6
	Farmed	13.21	6.78	12.14	4.54-33.59	1.89	32
Keta salmon (Chum salmon)	Wild	12.34	2.06	12.87	9.90-14.80	1.76	5
Pink salmon (Humpback salmon)	Wild	10.41	3.72	9.85	6.82-15.13	1.49	4
Rainbow trout	Farmed	10.71	5.46	8.57	7.11-20.35	1.53	5
Sea trout	Wild	17.31	3.42	16.86	14.14-21.40	2.47	4
Sockeye salmon (Red salmon)	Wild	24.87	8.84	13.25	16.39-34.39	3.55	6

Scombroidei							
Albacore tuna	Wild	9.81	2.29	9.35	7.82-13.29	1.40	5
Atlantic mackerel	Wild	34.35	17.05	23.25	17.48-52.08	4.91	7
Black marlin	Wild	9.82	2.86	8.30	7.41-14.35	1.40	5
Largehead hairtail (Beltfish)	Wild	14.02	1.35	14.40	12.53-15.15	2.00	3
Savalai hairtail (Ribbonfish)	Wild	12.56	0.32	12.53	12.25-12.90	1.79	3
Swordfish	Wild	15.15	4.87	16.50	7.11-19.60	2.16	5
Wahoo (Kingfish)	Wild	28.60	15.76	24.86	15.11-49.57	4.09	4
Yellowfin tuna	Wild	9.31	3.60	7.87	6.04-15.99	1.33	6
Scorpaeniformes							
Gurnard	Wild	30.36	15.95	25.98	17.77-66.43	4.34	8
Redfish (Norway redfish)	Wild	11.50	2.38	11.99	8.42-13.62	1.64	4
Stromateoidei, Anabantoidei							
Silver pomfret	Wild	5.35	0.99	5.40	4.12-6.48	0.76	4
Squaliformes							
Picked dogfish (huss)	Wild	26.29	8.37	30.45	16.65-31.76	3.76	3
Zeiformes							
John dory	Wild	15.99	5.79	14.60	9.17-27.81	2.28	7
Oreo dory (Smooth oreo dory)	Wild	7.66	6.69	4.80	3.49-19.51	1.09	5
Zoarcoidei							
Atlantic wolffish	Wild	38.34	29.34	36.95	12.75-66.71	5.48	4
**SHELLFISH**							
Crustaceans							
American lobster	Wild	104.63	41.05	96.36	69.28-156.52	14.95	4
*Edible crab – whole	Wild	244.12	52.12	249.90	189.35-293.11	34.87	3
*Edible crab – brown meat	Wild	225.14	29.44	237.71	181.21-243.93	32.16	4
*Edible crab – white meat	Wild	55.39	15.25	52.38	41.87-71.92	7.91	3
King prawn	Farmed	6.50	1.57	6.73	4.57-7.97	0.93	4
Langoustines (Norway lobster)	Wild	341.68	110.76	360.02	206.48-440.17	48.81	4
Tiger prawn	Farmed	7.27	3.36	7.08	3.81-11.13	1.04	4

Supplementary Table S1 Cont.

Common name	Wild/ Farmed	Iodine (μg.100 g^{-1} flesh)				% RWI, 140g serving	*n*
		Mean	SD	Median	Range (min-max)		
**SHELLFISH Cont.**							
Bivalves							
Atlantic deep-sea scallop	Wild	6.19	0.54	6.36	5.59-6.63	0.88	3
Atlantic razor clam	Wild	26.85	18.86	15.67	11.02-50.60	3.84	5
*Blue mussels	Wild	157.61	86.60	153.23	82.51-241.45	22.52	4
Blue mussels	Farmed	104.82	43.92	86.86	75.92-169.64	14.97	4
*Chilean mussels	Farmed	129.56	8.84	129.27	119.77-139.94	18.51	4

Clams	Wild	82.58	7.89	83.06	74.46-90.23	11.80	3
*Common edible cockle	Wild	126.89	20.67	127.88	104.86-146.92	18.13	4
Green-lipped mussels	Farmed	70.56	13.69	69.85	59.13-84.12	10.08	4
King scallop (+ roe)	Wild	18.90	7.27	19.06	11.54-26.08	2.70	3
Pacific oyster	Farmed	63.11	19.22	54.89	49.37-85.07	9.02	3
Patagonian scallop	Wild	7.97	2.92	6.81	5.80-11.29	1.14	3

References

6 Scientific Advisory Committee on Nutrition (SACN). Statement on Iodine and Health, February 2014. Available online: https://assets.publishing.service.gov.uk/government/uploads/system/uploads/attachment_data/file/339439/SACN_Iodine_and_Health_2014.pdf (accessed on 10 March 2021).

28 Scientific Advisory Committee on Nutrition (SACN) and Committee on Toxicity (COT). *Advice on Fish Consumption: Benefits and Risks*; The Stationary Office: Norwich, UK, 2004; p. 222.
